# A faulty interaction between SOD1 and hCCS in neurodegenerative disease

**DOI:** 10.1038/srep27691

**Published:** 2016-06-10

**Authors:** Gareth S. A. Wright, Svetlana V. Antonyuk, S. Samar Hasnain

**Affiliations:** 1 Molecular Biophysics Group, Institute of Integrative Biology, Faculty of Health and Life Sciences, University of Liverpool, UK

## Abstract

A proportion of Amyotrophic lateral sclerosis (ALS) cases result from impaired mutant superoxide dismutase-1 (SOD1) maturation. The copper chaperone for SOD1 (hCCS) forms a transient complex with SOD1 and catalyses the final stages of its maturation. We find that a neurodegenerative disease-associated hCCS mutation abrogates the interaction with SOD1 by inhibiting hCCS zinc binding. Analogously, SOD1 zinc loss has a detrimental effect on the formation, structure and disassociation of the hCCS-SOD1 heterodimer. This suggests that hCCS functionality is impaired by ALS mutations that reduce SOD1 zinc affinity. Furthermore, stabilization of wild-type SOD1 by chemical modification including cisplatination, inhibits complex formation. We hypothesize that drug molecules designed to stabilize ALS SOD1 mutants that also target the wild-type form will lead to characteristics common in SOD1 knock-outs. Our work demonstrates the applicability of chromatographic SAXS when studying biomolecules predisposed to aggregation or dissociation; attributes frequently reported for complexes involved in neurodegenerative disease.

Mutation of superoxide dismutase-1 (SOD1) causes amyotrophic lateral sclerosis (ALS)^1^. SOD1-related ALS is a classical motor neuron disease; pathology is restricted to death of upper and lower motor neurons with cognitive impairment reported rarely. Denervation of the neuromuscular junction and motor neuron loss leads to progressive muscle weakness, paralysis and ultimately death, most frequently through respiratory failure. The molecular characteristics of this proteopathy proceed from mutant SOD1 structural instability to unfolding and aggregation^2^,^3^. This pathological chain of events begins in the non-symptomatic phase of the disease^4^,^5^.

The nascent SOD1 polypeptide must be extensively post-translationally modified. Sequential zinc binding, dimerization, copper acquisition and formation of a disulphide bond contribute to SOD1 activation and thermal stability^6^. Zinc binding and disulphide tethering of loop IV increases the thermal stability of wild-type SOD1 dramatically, shifting its unfolding transition from body temperature for some ALS mutants to 75 °C for the fully mature wild-type enzyme^7^. High stability ensures high concentrations of soluble protein can be maintained. As a result, superoxide anions are more likely to encounter SOD1 and begin the detoxification process by conversion to hydrogen peroxide. Hindering the SOD1 maturation pathway would however provide much material with a propensity for toxic unfolding. This is observed as characteristic SOD1 aggregates in the neural tissue of ALS sufferers^8^,^9^,^10^. Work with transgenic model organisms indicates these inclusions are composed of metal depleted, disulphide reduced SOD1^11^,^12^,^13^. A faulty maturation pathway seems to be the cause of SOD1-ALS.

The central facilitator of SOD1 maturation is the multi-domain copper chaperone for SOD1, hCCS, a predominantly homodimeric enzyme whose central domain bears a high degree of sequence identity with SOD1^14^. Both localize in the cytosol and the mitochondrial intermembrane space (IMS). SOD1 disulphide oxidation and copper transfer are catalysed by hCCS in the cytosol and during SOD1 import into the IMS^15^,^16^,^17^. Mitochondrial accumulation of mutant SOD1 has been suggested to be an important cause of motor neuron pathology and death^18^. hCCS structural modularity means its functions are not linked^19^ but the efficiency of disulphide and copper transfer are dependent on the formation of an heterodimeric complex with SOD1 through its zinc-binding, SOD1-like domain (D2)^20^. The relative concentration of hCCS is low with respect to SOD1^21^. Thus one molecule of hCCS is believed to interact with several SOD1 molecules through a series of heterodimer complex formation and dissociation events perhaps at the membrane-solvent interface^22^. Mutation of hCCS has been predicted to cause its aggregation^23^ and hCCS can be found with SOD1 in cytoplasmic inclusions^8^,^10^. However there were no accounts of human, disease-causing mutations until the discovery of an R163W missense substitution in an individual with system-wide and fatal neurological abnormalities in 2012^24^.

Here we describe how pathogenic SOD1 and hCCS mutations that inhibit zinc binding render normal complex formation and disassociation ineffective. hCCS knock-out SOD1 mutant transgenic mice show symptoms indistinguishable from their hCCS expressing litter mates^25^. Our work indicates this is because mutant SOD1 inhibits hCCS function. This forces a reappraisal of the role of hCCS in SOD1 ALS. We have reconstructed the structure of the hCCS-SOD1 heterodimer using small angle X-ray scattering data taken from heterogenous solutions of hCCS, SOD1 and hCCS-SOD1 collected after separation by analytical size exclusion chromatography. We found that structural changes to SOD1 present in the metal deficient SOD1 homodimer are also present when complexed with hCCS. In addition, we observed that heterodimer complexation is inhibited by chemical modification of SOD1 with drug-like molecules known to stabilize SOD1 by targeting Cys111 including cisplatin^26^,^27^. These findings fit an important piece in the puzzle of broken SOD1 maturation in ALS and pose a salutary caution to drug discovery efforts that aim to stabilize SOD1 for therapeutic benefit. It is important to ensure that stabilization does not alter complex formation with hCCS and its subsequent disassociation.

## Results

### R163W hCCS recapitulates the characteristics of ALS mutant SOD1

**Figure 1:**
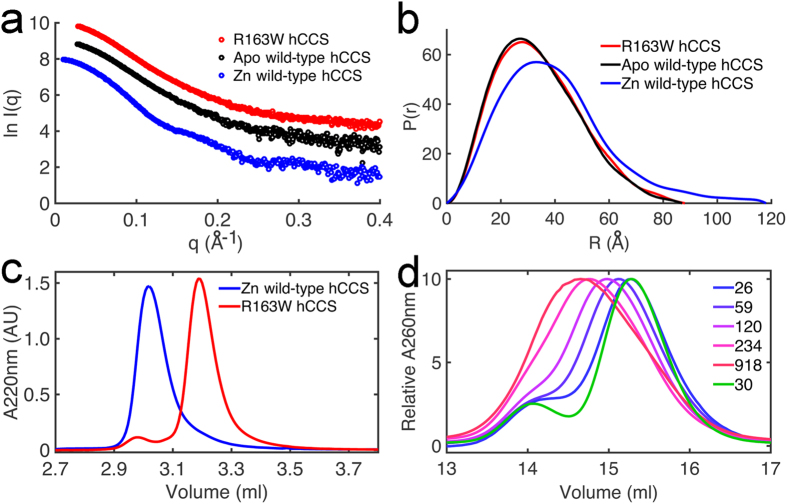
R163W hCCS is monomeric and unable to form a heterodimer with SOD1. (**a**) SAXS from R163W hCCS in comparison with Zn-wild-type and metal free hCCS. R163W hCCS radius of gyration is 24.9 Å (0.66 < q*R*_g_ < 1.27) and *D*_max_ 85 ± 5 Å in comparison with 31.3 Å and 118 ± 5 Å for Zn-wild-type^10^ and 26.4 Å and 87 ± 5 Å metal apo-wild-type. (**b**) Distance distribution function of R163W hCCS and metal apo wild-type hCCS. χ^2^ R163W 0.88, apo wild-type 0.93. Zn wild-type 0.94. Data used q < 0.4 Å^−1^. Curves normalized by area. (**c**) SEC showing predominantly monomeric R163W hCCS in comparison with dimeric wild-type. The molecular masses of R163W species were calculated by multi-angle laser light scattering to be 29.1 kDa ± 4% for the species at 3.19 ml (monomer) and 55.6 kDa ± 10% for the species at 2.98 ml (dimer). SEC performed on an Agilent BioSEC-3 column. n = 3. (**d**) SEC showing concentration dependent dimerization of R163W hCCS. Monomeric R163W (15.3 ml) shifts to dimer (14.6 ml) with increasing concentration (*blue* to *red*) and moves back to monomer after dilution to initial concentration (green). SEC performed on a GE Superdex 200 10/300 column. Concentrations (μM) are shown in the legend and refer to the protein concentration before application to the SEC column.

Initial SAXS analysis indicated wild-type and R163W hCCS have very different structures. R163W however has similar overall scattering pattern and size parameters when compared with metal apo-hCCS (Fig. 1a,b). Arg163 is found beside zinc co-ordinating His164 in the upper zinc loop. We postulated the substitution would be detrimental to zinc binding and confirmed this by ICP-MS analysis; wild-type hCCS was found to contain stoichiometric amounts of zinc (1.0 zinc ions/monomer) where-as R163W hCCS contained 1.0 zinc ion/13.1 monomers. Wild-type hCCS forms a stable dimer when zinc metalated^14^ but zinc removal shifts the equilibrium in favour of monomer^28^. Analysis of R163W hCCS by size exclusion chromatography shows the protein is predominantly monomeric (Fig. 1c). R163W hCCS is however able to form a majority dimer at high concentrations (Fig. 1d). This is reversible upon dilution indicative of a shift in the monomer-dimer equilibrium towards the monomer (Fig. 1d).

**Figure 2:**
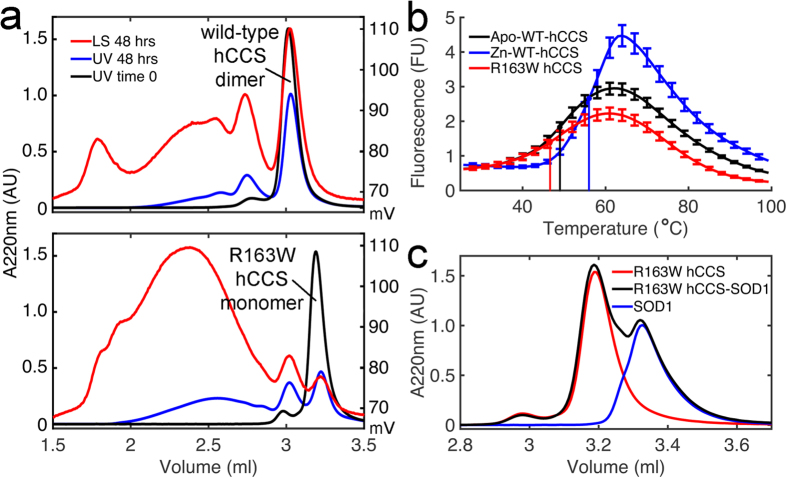
R163W hCCS recapitulates the molecular characteristics of ALS mutant SOD1 and cannot form a complex with SOD1. (**a**) *Upper* - SEC showing zinc metalated wild-type hCCS aggregation. 34% wild-type dimer (2.9–3.15 ml) is lost to aggregation (1.7–2.9 ml) after 48 hours in comparison with *Lower* - 63% of R163W monomer (3.1–3.35 ml). After 24 hours 10 and 16% of wild-type dimer and R163W monomer are lost to aggregation respectively (data not shown). Increased static light scattering at 90^o^ (red chromatograms) over elution volume around 2.3 ml indicates the presence of more high molecular mass species for R163W. n  =  3. (**b**) DSF showing that R163W hCCS is thermally unstable. At 26 °C Zn-wild-type hCCS, apo-wt hCCS and R163W hCCS exhibit equal reporter fluorescence indicating all three proteins share comparable hydrophobic residue exposure. R163W and apo-wt hCCS begin to unfold as soon as the temperature starts to rise. This contrasts Zn-wild-type hCCS, which shows no increase in fluorescence until approximately 45 °C signifying resistance to thermal unfolding. n = 8 with standard deviation for 1 in 3 data points and mid-point melting temperature plotted. (**c**) SEC indicating that R163W hCCS cannot heterodimerize with SOD1. A 1:1 stoichiometric mixture of R163W hCCS and SOD1 does not shift the component proteins from their respective monomeric (3.19 ml) and homodimeric (3.33 ml) positions. n = 3.

Incomplete SOD1 metalation impedes acquisition of structural stability. Unfolding and exposure of hydrophobic centres ensues^29^ with spontaneous formation of high molecular mass oligomers at physiological temperature^30^. Analogously, R163W hCCS is more prone to aggregation than wild-type hCCS under physiological conditions (Fig. 2a). In our assay, wild-type hCCS retained more than half of the dimeric starting material with reduced appearance of large aggregates in comparison with R163W hCCS. In addition, R163W hCCS is also unable to form tetramers (Fig. 2a, 2.75 ml). Aggregation propensity and structural instability are linked by thermal instability. The mid-points of thermally induced unfolding transitions for Zn-wild-type, apo-wild-type and R163W hCCS were found to be 56.0 ± 0.5, 49.0 ± 0.8 and 46.6 ± 0.5 °C respectively (Fig. 2b). Therefore, R163W hCCS thermal instability exceeds that of zinc demetalated wild-type hCCS.

SOD1 copper acquisition and disulphide reduction by hCCS are dependent on formation of an heterodimeric complex mediated by the hCCS central domain^20^. This heterodimer is of intermediate molecular mass^31^. Ablation of domain two (D2) zinc binding prevents complexation with SOD1^28^,^32^. Similarly, when R163W hCCS is presented with reduced SOD1 there is no discernible complex formation (Fig. 2c). This provides a clear explanation for the lack of SOD1 activity observed in R163W hCCS homozygous fibroblasts^24^.

### hCCS-SOD1 heterodimer structure and the effect of SOD1 zinc loss

**Figure 3:**
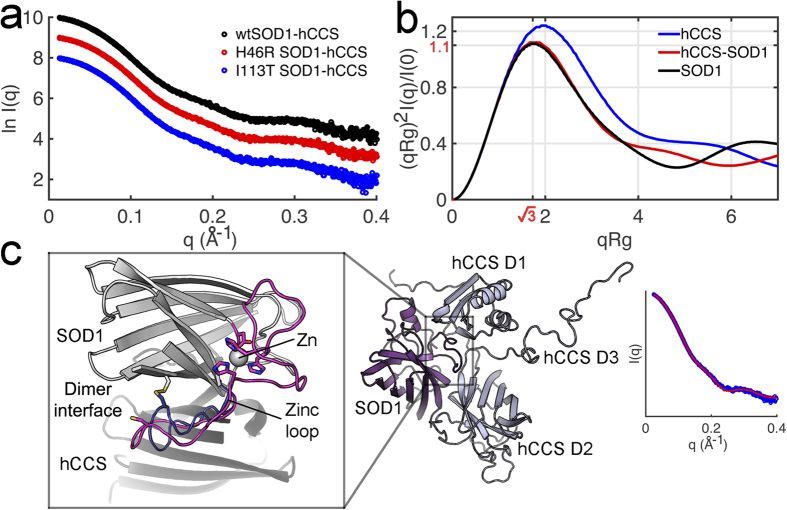
ALS-SOD1 mutants form a compact heterodimer with hCCS. (**a**) Small angle X-ray scattering by ZnSOD1-ZnCCS. Wild-type, H46R and I113T–hCCS heterodimer complexes have superimposable scattering patterns and indistinguishable size parameters; *R*_g_ 24.8 ± 0.3 Å, *D*_max_ 78 ± 6 Å, n = 8. Mutant and wild-type SOD1-hCCS complexes co-elute from SEC indicating identical hydrodynamic radii (data not shown). (**b**) The hCCS-SOD1 heterodimer has a conformationally rigid, globular structure. Dimensionless Kratky plot of X-ray scattering from hCCS, SOD1 and the hCCS-SOD1 heterodimer indicates the conformational flexibility displayed by the hCCS homodimer is lost when complexed with SOD1. A folded protein will have a peak maximum at √3 and 1.1. Increased conformational flexibility pushes the peak further into the positive section of the graph, quadrant I, as can be seen for homodimeric hCCS. In contrast, ZnSOD1-hCCS does not show any more conformational plasticity than the Zn-SOD1 homodimer. (**c**) *Centre* – Structure of Zn-wtSOD1-hCCS heterodimer modelled against experimental SAXS data. *Right* – Agreement of SAXS data (blue) with computed model scattering pattern (red), χ^2^ 1.5 and volatility ratio (*V*_*R*_)^68^ 0.37 over 0.0242 < q < 0.5 Å^−1^. Fitting residuals are shown in Supplementary Figure S4. *Left* – The SOD1 zinc loop *in situ* at the SOD1-hCCS dimer interface. *Blue* shows the position of the disulphide bonded loop as found in the SOD1 homodimer and *magenta* shows the loop’s position in our model in the disulphide reduced state indicating conformational mobility and changes to the dimer interface.

For hCCS to function correctly the hCCS-SOD1 heterodimer must be a transitory complex. *In vitro* however the complex can be perpetuated by inhibiting SOD1 disulphide formation^31^. This allows time to study its structure. Zinc metalated ALS mutant and wild-type SOD1 form indistinguishable heterodimers with hCCS when observed by small-angle X-ray scattering (Fig. 3a). hCCS-SOD1 forms a more compact structure in comparison with the hCCS homodimer as evident in the SAXS data (Fig. 3b). Furthermore, the stability deficit conferred by ALS mutations is removed when SOD1 is complexed with hCCS (Supplementary Fig. 1). Modelling the heterodimer against the SAXS data in Fig. 3a shows the copper binding Atx1 domain closely associated with SOD1 and movement of the upper zinc loop without the disulphide bond constraint (Fig. 3c).

**Figure 4:**
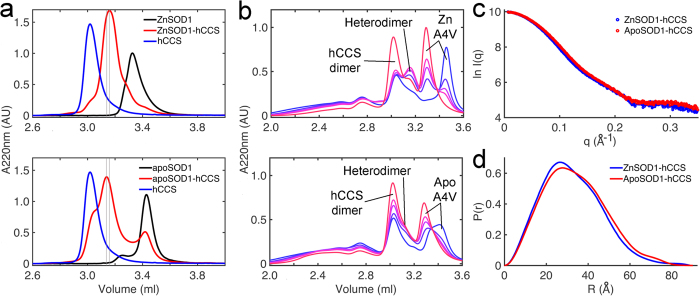
Heterodimer formation and structure is disturbed by SOD1 zinc loss. (**a**) *Upper* - Size exclusion chromatogram of zinc wild-type SOD1-hCCS heterodimer (3.16 ml) formation under reducing conditions. The reaction goes to completion with little homodimeric SOD1 or hCCS remaining. n = 3. *Lower* - The complexation reaction between zinc deficient wild-type SOD1 and hCCS does not go to completion. Homodimeric hCCS and apo-SOD1 remain in solution (3.02 and 3.42 ml respectively). Elution of the hCCS-SOD1 complex is also shifted to 3.14 ml indicating increased hydrodynamic radius. n = 3. (**b**) *Upper* - SEC time course of zinc A4V SOD1-hCCS heterodimer complex formation separated by SEC under non-reducing conditions (*blue* to *red* chromatograms show 8 minutes to 4 hours after complexation). The A4V SOD1 heterodimer forms then disappears as the SOD1 cysteine thiols are oxidized to form the disulphide and homodimeric SOD1 is evolved. In comparison with, *Lower* - evolution and subsequent disassociation of apo-A4V SOD1-hCCS. A small amount of heterodimer is observed between elution of homodimeric hCCS and SOD1 at 3.16 ml and much less SOD1 homodimer is evolved. (**c**) SAXS curves for zinc and apo wild-type SOD1-hCCS heterodimer. (**d**) P(r) function of c showing significant real-space conformational differences. Data used: 0.022 < q < 0.4 Å^−1^, *D*_max_ ZnSOD1-hCCS 90 Å, apoSOD1-hCCS 89 Å. ApoSOD1-hCCS χ^2^ 0.91, ZnSOD1-hCCS χ^2^ 1.01. Curves normalized by area.

**Figure 5:**
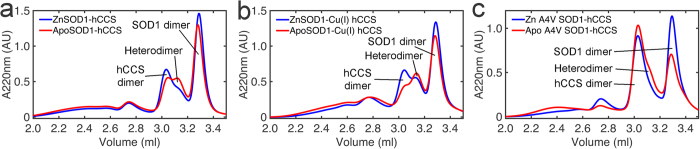
Heterodimer disassociation is inhibited by SOD1 zinc loss. (**a**) SEC showing dissociation of copper apo/zinc holo hCCS-SOD1 heterodimer after 20 hours of incubation following initial complexation. *Red* – metal apo SOD1-hCCS, *Blue* – Zinc metalated SOD1-hCCS. Less heterodimer and more SOD1 homodimer can be observed when zinc metalated SOD1 dissociates from hCCS. (**b**) As above, but hCCS domain I is copper metalated. (**c**) As above with A4V mutant SOD1. The amount of apoA4V SOD1-hCCS heterodimer remaining is more than for the zinc metalated form and the amount of dimeric SOD1 evolved is less.

Zinc metalation is thought to be the first step in the succession of SOD1 post-translational modification (PTM) events^16^ however weakened affinity for zinc is a common trait of ALS SOD1 mutants^33^,^34^,^35^,^36^,^37^,^38^. Apo-SOD1 is able to form an heterodimeric complex with Zn-hCCS^20^. When apo-SOD1 complexes with hCCS several important differences are observed: Complex formation does not go to completion; hCCS and apo-SOD1 homodimers remain in the solution (Fig. 4a,b) and the hydrodynamic radius (*R*_h_) of the complex increases as evidenced by a shift in its elution position from SEC. The SAXS profile changes and the radius of gyration (*R*_g_) increases from 24.8 to 26.2 Å (Fig. 4c,d). Heterodimer complex disassociation is inhibited regardless of the copper loading of hCCS or ALS mutations of SOD1 (Fig. 5). This clearly demonstrates that SOD1 zinc loss induces structural and functional abnormalities in the heterodimer formed with hCCS. These changes are comparable with those found in the immature, zinc deficient SOD1 homodimer (Supplementary Figs 2 and 3) and are indicative of a loosening of the compact protein structure^39^.

### Stabilizing chemical modifications inhibit heterodimer formation

**Figure 6:**
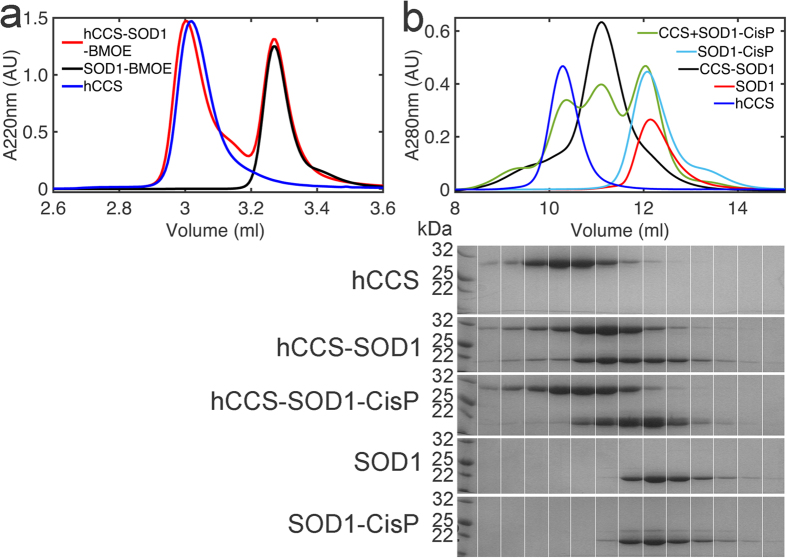
hCCS-SOD1 complex formation is inhibited by exogenous covalent stabilization of the SOD1 dimer. (**a**) Covalent tethering of SOD1 monomers by addition of BMOE to opposing Cys111 residues completely inhibits heterodimerization with hCCS as observed by SEC. n = 3. (**b**) *Upper* - Addition of cisplatin to SOD1 Cys111 inhibits heterodimer formation with hCCS. The curves shown are representative of three chromatograms. *Lower*-SDS-PAGE of 0.5 ml fractions taken from the SEC above showing reduced SOD1-hCCS complex formation after SOD1 Cisplatin binding. It is also clear from the SOD1 and SOD1-CisP SDS-PAGE that cisplatin does not cause covalent tethering of the SOD1 dimer.

Stabilizing SOD1 by binding small molecules to specific sites that mediate SOD1 toxicity has shown promise as a strategy to develop targeted therapeutics^26^,^27^,^40^. Crosslinking opposing SOD1 monomers offers the potential to permanently inhibit dimer disassociation and greatly increases the protein’s thermal stability^26^. However, complex formation with hCCS necessitates SOD1 homodimer disassociation and covalent dimer tethering with Bismaleimidoethane (BMOE) completely inhibits heterodimer formation (Fig. 6a). Covalent addition of cisplatin does not crosslink opposing monomers but has been shown to increase SOD1 stability and strongly disfavour aggregation^27^. In this case heterodimer formation is inhibited but not completely (Fig. 6b) and may reflect the 50% occupancy of cisplatin in a SOD1 dimer^27^.

## Discussion

Proteins interact with their surroundings throughout their lifetime. This decides their location, function, the timing and means of their degradation or any pathogenic effects. The efficacy of these interactions is therefore very important. Retrieving structural information from these biomacromolecular conjugates is however often complicated by their presence in heterogeneous states; conformational plasticity, oligomeric equilibria, aggregation and degradation for example. The case in point, the hCCS-SOD1 complex, has been intensely studied since the discovery of CCS but has been refractory to structural characterization due to its size, transient nature and the difficulty of isolating it in pure form. Small angle X-ray scattering coupled with a size exclusion chromatography stage allows one to pick small tranches of data from otherwise polydisperse solutions^14^. Here we have been able to create a model of the complete, wild-type human CCS-SOD1 heterodimer using data taken from a solution otherwise contaminated by uncomplexed constituents.

Understanding of SOD1-related ALS has grown with observations of the mutant protein *in silico*, *in vitro*, cell and animal models, and histopathology of post-mortem CNS tissue. The coherent theme across SOD1 studies is that structural instability resulting from disease-causing mutations promotes unfolding and monomerization. A symptom of this process is aggregation of SOD1 within effected motor neurons and associated tissues^41^, denervation at neuromuscular junctions and nerve death. Conversely, wild-type SOD1 is a perfect example of evolutionary bioengineering; small, compact, structurally solid and highly active. Zinc binding and disulphide bonding synergize to lock the protein’s conformation. SOD1 loop IV houses all the zinc co-ordinating amino acid side chains, a cysteine involved in disulphide bonding and forms part of the SOD1 dimer interface. This long loop shows a high degree of conformational freedom that reduces homodimer affinity when zinc and the disulphide are absent^42^. Zinc bound, disulphide reduced SOD1 is the normal substrate for hCCS. In this state half of loop IV is tethered and conformational flexibility around Cys57 is limited. This form must have a higher affinity for hCCS than itself. Disulphide transfer anchors loop IV and forms the stable dimer interface. This shifts the affinity of SOD1 from hCCS to favour homodimerization. In this final act copper can be donated to SOD1 creating the active and stable enzyme capable of performing its biological function of dismutation of superoxide.

Here we have shown reduced metalation resulting from the R163W neurodegenerative disease-associated mutation in hCCS; a situation analogous to the diminished zinc affinity of ALS-SOD1 mutants. The R163W substitution prevents hCCS forming the critical heterodimer with SOD1 (Fig. 2c), greatly reduces SOD1 activity and triggers unfolded protein response markers^24^. Several other lines of evidence also indicate that the hCCS-SOD1 interaction is effected by disease causing mutations: 1) hCCS overexpression in a G93A SOD1 mouse causes extreme ALS-like symptoms and drastically reduces life-expectancy^43^, 2) hCCS over-expression increases the proportion of disulphide oxidized wild-type SOD1 but not ALS mutant SOD1^44^, 3) SOD1 is found to be metal depleted and disulphide reduced in the inclusions found in mutant SOD1 transgenic animal models^11^,^12^,^13^. 4) SOD1 activity is lower in tissues from SOD1-ALS sufferers^3^,^45^. Activity may be reduced by proteolysis, aggregation or inability to copper metalate, indeed SOD1 copper loss leads to aggregation which correlates with disease severity^46^, but each characteristic is indicative of heterodimer malfunction given hCCS’s dual function.

Our observations of the complex formed between SOD1 and wild-type hCCS confirm the notion of a faulty interaction in ALS. Each step in the homodimer-heterodimer-homodimer cycle requires changes to the SOD1 dimer interface and the efficacy of each step is determined by SOD1 zinc metalation. Heterodimer formation between hCCS and zinc-SOD1 is fast and complete under reducing conditions but complexation is inhibited by SOD1 zinc loss (Fig. 4a,b). For wild-type homozygous individuals this delicate balance of affinities prioritises zinc loaded SOD1 for interaction with hCCS. This distinction must be made because copper acquisition in the absence of zinc yields a catalytically inactive enzyme^47^. However, ALS SOD1 mutations reduce the affinity for zinc^33^,^34^,^35^,^36^,^37^,^38^. The high cellular chelation capacity will divert zinc to other destinations leaving mutant SOD1 zinc free and with reduced potential for hCCS catalysed disulphide formation.

The portion of metal deficient SOD1 that does complex with hCCS has a structural perturbation that inhibits its dissociation (Fig. 4a,c,d). We propose that increased loop IV mobility in the absence of SOD1 zinc slows catalysis of disulphide transfer by hCCS and increases the life-time of the heterodimer complex. Also, copper transfer to SOD1 cannot take place under these circumstances^20^. hCCS recycling would be prevented when metal deficient SOD1 is unable to relinquish its chaperone. Nascent SOD1, whether wild-type or mutant, then has a reduced chance of forming a complex with hCCS. The susceptibility of mutant SOD1 to disulphide reduction will exacerbate this effect^48^,^49^ as will increasing the amount of mutant SOD1, as in the case of transgenic mouse models^25^. These situations effectively create an hCCS functional knock-out by further depleting the pool of available hCCS; a scenario that provides material for accumulation of small toxic oligomers^50^,^51^, aggregate growth^13^ and cell to cell propagation^52^ by hindering SOD1 maturation. It is also interesting to note that heterodimer formation with hCCS alleviates the destabilizing effect of ALS mutations (Supplementary Fig. 1). Increasing the amount of hCCS and mutant SOD1 creates an abundance of long-lived, thermally stable complexes. This may explain the lack of SOD1 misfolding and aggregation observed in SOD1 transgenic mouse and cell models where hCCS is overexpressed^43^,^44^,^53^.

Stabilizing the zinc bound, disulphide-reduced form of SOD1 by chemical modification prevents complexation with hCCS (Fig. 6). This would inhibit the chaperone’s functions^20^. Blocking the route of copper to SOD1 through hCCS has been shown to inhibit cancer proliferation^54^. We note that inhibiting the direct interaction of SOD1 with its chaperone may indeed be the contributory factor in the success of cisplatin as a cancer therapeutic. SOD1 knock-out does not cause ALS but does lead to denervation in a manner similar to the normal aging process and a host of other complications. This presents a problem if we are to stabilize SOD1 for therapeutic benefit in ALS. Cys111 modification is currently the only valid non-native strategy to stabilize SOD1. What remains to be seen is the effect of stabilization at alternative sites. It is clear however that any perturbation of the affinities that regulate complex formation and disassociation would negatively affect both SOD1 disulphide formation and copper activation. A stabilizing molecule that specifically targeted the mutant protein without binding the wild-type form would circumvent this problem. However more tractable options may include prevention of the interactions that lead to aggregation or stimulation of hCCS mediated SOD1 maturation. Diacetyl-bis(4-methylthiosemi-carbazonato)copper^II^ uses the latter route along the CtrI-hCCS-SOD1 copper trafficking pathway to stabilize intracellular SOD1 and improve transgenic SOD1 mouse survival^55^,^56^,^57^. This underscores both the role of hCCS in ALS but also its potential for therapeutic intervention.

## Materials and Methods

### Protein production, oligomeric state, aggregation and thermal stability

SOD1 and hCCS recombinant proteins were produced as described previously^14^,^40^. Metal apo proteins were produced as described^58^,^59^,^60^ and metal ion content was determined by ICP-MS where applicable. Protein concentrations were determined by absorbance at 280 nm using monomer molar extinction coefficients. R163W concentration dependent dimerization was observed on a Superdex 200 10 300 column with soluble phase flow 0.5 ml/min in 20 mM Tris-HCl, 150 mM NaCl (TBS) with 1 mM DTT. 500 μl sample was loaded at concentrations ranging from 26 to 918 μM. The high concentration protein was then diluted back down to 30 μM. SOD1-hCCS complex formation was achieved by overnight reduction of SOD1 with 40 mM dithiothreitol (DTT) at 4 °C and mixing at a 1:1 molar ratio with hCCS before SEC. SEC was then performed at 20 °C on an Agilent 1260 series HPLC using an Agilent BioSEC-3 4.6 × 300 mm column with 3 μm bead size and 300 Å pore size at a soluble phase flow rate of 0.4 ml/min in TBS with 1 mM DTT unless otherwise stated. Complex dissociation experiments were performed after SOD1 reduction and desalting into oxygen purged TBS. hCCS was copper loaded with tetrakis(acetonitrile)copper(I) hexafluorophosphate under anaerobic conditions followed by desalting. hCCS loaded with copper (I) had 87% occupancy as determined by ICP-MS. Reactions were incubated at 20 °C over a time-course then separated by SEC. To find the relative aggregation propensity of R163W hCCS both wild-type and mutant were incubated at 37 °C over a 48 hour time-course. Samples were taken at 0, 24 and 48 hours then separated by SEC using UV absorption and static light scattering (658 nm) to monitor protein aggregation. Protein thermal stability was determined by differential scanning fluorometry. In each case, fluorescence (Ex: ~470 nm , Em: ~610 nm) was measured from 10 μM protein with 40× Sypro-orange dye heated at 1 °C min^−1^ from 25 to 95 °C. Melting temperatures were defined as the temperature at half peak height of the unfolding transition using SimpleDSFviewer^59^.

### Small-angle X-ray scattering and model building

Small angle X-ray scattering measurements were performed using the SEC-SAXS apparatus at beamline SWING^60^, Synchrotron Soleil. Acquisition of SOD1, hCCS and hCCS-SOD1 complex data has been described previously^14^,^61^. R163W SAXS data were collected after separation of 6 nmoles protein on an Agilent BioSEC-3 4.6 × 300 mm column with 3 μm bead size and 100 Å pore size. Distance distribution functions for ZnSOD-hCCS and apoSOD1-CCS were calculated using data 0.02 < q < 0.4 Å, D_*max*_ 89 and 90 Å, χ^2^ 1.00 and 0.96 respectively using GNOM^62^ and ScÅtter. Initial models of wild-type zinc loaded hCCS monomer were generated by I-Tasser from the primary sequence including cloning fragment^63^. The initial orientation of the SOD1-hCCS domain II interface was achieved by substituting the crystal structure of hCCS D2 (1DO5)^64^. This was followed by alignment of an human SOD1 monomer (2C9V)^65^ to the opposing hCCS D2 monomer. Five heterodimers were constructed in this fashion using five output models from I-Tasser. The model with lowest χ^2^ (4.93) against experimental data was used in subsequent steps. Disulphide bonds were removed and disulphide loops designated as flexible (SOD1 amino acids 50–62, hCCS 134–146). The positions of domains and loops were then modelled by torsion angle molecular dynamics (MD) using CNS with the SOD1-hCCS domain II interface locked^66^. MD simulations were performed at 300,000 K, after energy minimization, without simulated annealing. The procedure began with 40–80 ps simulations with infrequent sampling. When modelling coalesced around an arrangement of domains, 1–10 ps simulations with frequent sampling were used to optimize the structure. At each stage models were compared to experimental data using FoXS^67^.

### Complexation with chemically modified SOD1

BMOE and cisplatin were covalently bonded to SOD1 using the protocols of Auclair *et al*.^26^ and Banci *et al*.^27^ respectively. SOD1 was reduced and complexed with hCCS as above with the exception that 100 nmoles of hCCS and SOD1 with or without cisplatin bound were separated by SEC on a Superdex 75 10 300 column and fractions taken for analysis by reducing SDS-PAGE.

## Additional Information

**How to cite this article**: Wright, G. S. A. *et al*. A faulty interaction between SOD1 and hCCS in neurodegenerative disease. *Sci. Rep*. **6**, 27691; doi: 10.1038/srep27691 (2016).

## Supplementary Material

Supplementary Information
