# Supplementary material for: A faulty interaction between SOD1 and hCCS in neurodegenerative disease
Source: Sci Rep. 2016 Jun 10;6:27691. doi: 10.1038/srep27691 (PMC4901319; doi:10.1038/srep27691)

Supplementary Information:

A faulty interaction between SOD1 and hCCS in neurodegenerative disease

Gareth S.A. Wright[1,2], Svetlana V. Antonyuk[1] & S. Samar Hasnain[1,2]
[1] Molecular Biophysics Group, Institute of Integrative Biology, Faculty of Health and Life Sciences, University of Liverpool, UK.
[2] To whom correspondence should be addressed.

Supplementary Figure S1. DSF showing that SOD1 instability resulting from ALS mutations is largely ameliorated by complexation with hCCS. Unfolding transitions are I113T SOD1 44.4 ± 0.5, wtSOD1 51.5 ± 0.5, I113T SOD1- hCCS 52.4 ± 0.7 and wtSOD1-hCCS 53.3 ± 0.5 ^{o}C.

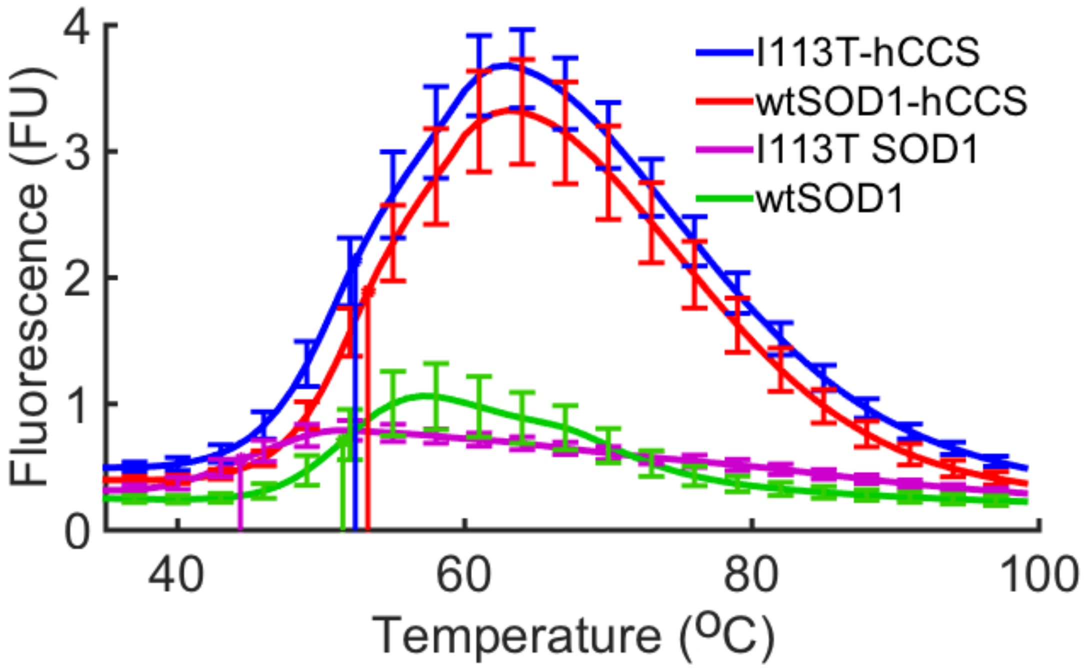

Supplementary Figure S2. Size exclusion chromatograms of zinc metalled and zinc free wild-type SOD1 showing apo-SOD1 eluting before zinc-SOD1 indicating increased R_h.

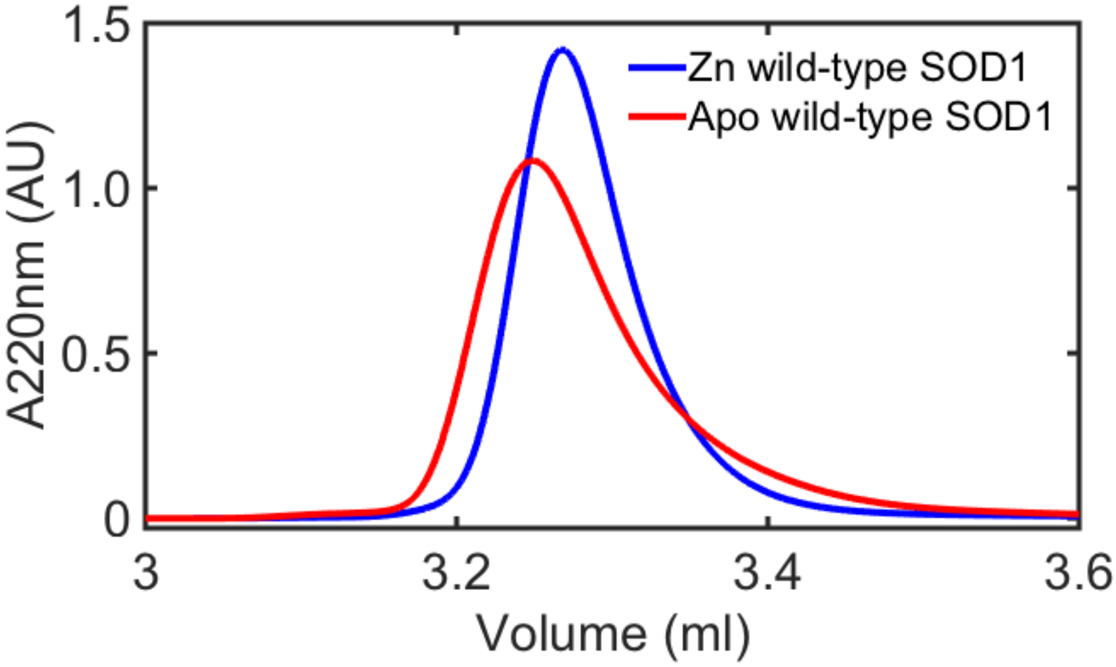

Supplementary Figure S3. SAXS curves for zinc metalled and zinc free wild-type SOD1. Zn-SOD1 R_g 20.9, apo-SOD1 R_g 21.9, D_{max} 65 Å.

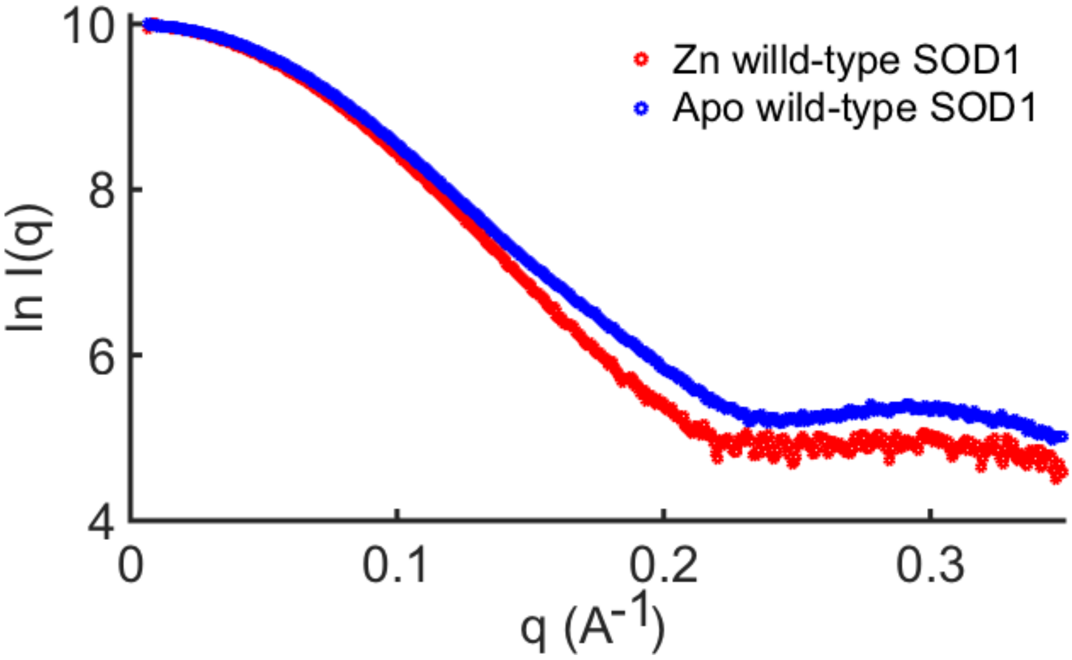

Supplement: Supplementary Information [file srep27691-s1.pdf]
